# Proteomic Techniques to Examine Neuronal Translational Dynamics

**DOI:** 10.3390/ijms20143524

**Published:** 2019-07-18

**Authors:** Shon A. Koren, Drew A. Gillett, Simon V. D’Alton, Matthew J. Hamm, Jose F. Abisambra

**Affiliations:** Department of Neuroscience and Center for Translational Research in Neurodegenerative Disease, University of Florida, Gainesville, FL 32601, USA

**Keywords:** nascent proteomics, ribosome, neurodegeneration, translation, Alzheimer’s disease

## Abstract

Impairments in translation have been increasingly implicated in the pathogenesis and progression of multiple neurodegenerative diseases. Assessing the spatiotemporal dynamics of translation in the context of disease is a major challenge. Recent developments in proteomic analyses have enabled the resolution of nascent peptides in a short timescale on the order of minutes. In addition, a quantitative analysis of translation has progressed in vivo, showing remarkable potential for coupling these techniques with cognitive and behavioral outcomes. Here, we review these modern approaches to measure changes in translation and ribosomal function with a specific focus on current applications in the mammalian brain and in the study of neurodegenerative diseases.

## 1. Introduction

RNA translation is a dynamic process that regulates many baseline cellular functions and adaptations in response to environmental cues. Studies in the early 1960s demonstrated the involvement of protein synthesis in memory formation and recall, thereby providing evidence that translation links mechanisms at the cellular level to complex brain function [1,2,3]. As techniques developed, further evidence suggested that translation plays a crucial role in synaptic plasticity and cognition beyond maintaining steady-state protein levels [4,5,6]. An emerging theory of local protein synthesis at axons and dendrites also identifies the role of protein synthesis in regulating the spatial and temporal dynamics of neurite outgrowth, synaptic release, and plasticity, with implications in multiple neurological disorders and neurodegenerative diseases [7,8,9,10]. Furthermore, since neurons are especially vulnerable to proteostatic damage due to their unique cellular architecture and increased activity, translation assists in maintaining neuronal function and health in response to stressful or toxic conditions [11,12,13,14,15]. Since the human brain contains approximately 17,000 protein-coding genes and neurodegenerative diseases exhibit nearly 2000 proteins with altered protein expression [16], investigations into the role of altered translation are crucial to better understand these diseases. Together, these studies, among many others, emphasize translation as a crucial pathway for investigating neuronal adaptations to stress and disease and synaptic plasticity.

Protein synthesis in eukaryotes relies on a complex network of coordinating mechanisms that facilitate RNA translation and ribosome function (reviewed in [5,6]). Modern techniques can investigate translation from the deep sequencing of ribosome-associated RNA fragments (RiboSeq) to the proteomic analysis of nascent peptides (reviewed in [17]). Sequencing-based methods employ the amplification capacity of RNA, enabling the analysis of small amounts of input samples. These current sequencing strategies achieve an unsurpassed resolution of translational dynamics at the nucleotide level [18]. However, RNA transcripts may be associated with the ribosome, albeit not actively translated, thus potentially confounding RiboSeq results. Furthermore, other post-transcriptional mechanisms such as those that involve micro RNAs (miRNA) or non-coding RNAs (ncRNA) may alter levels of translation which might not be identified using typical ribosome sequencing strategies [19].

**Figure 1:**
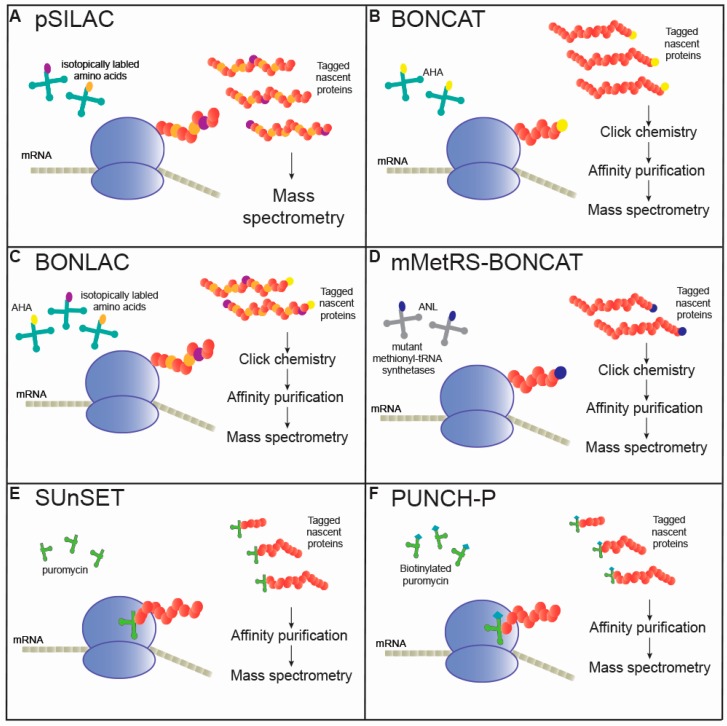
Overview of nascent proteomic techniques. (**A**) pSILAC: Pulsed stable isotope labeling with amino acids in cell culture. (**B**) BONCAT: Bio-orthogonal non-canonical amino acid tagging. (**C**) BONLAC: Combination of BONCAT and pSILAC. (**D**) mMet-BONCAT: Bio-orthogonal non-canonical amino acid tagging with expanded, mutant methionyl-tRNA synthetases. (**E**) SUnSET: Surface sensing of translation. (**F**) PUNCH-P: Puromycin-associated nascent chain proteomics.

Accordingly, several techniques to analyze translation at the protein level have been developed, and these can be paired with mass spectrometry (MS) analysis of the whole proteome to correlate an altered translation with total protein levels [20]. These nascent proteomic techniques typically involve the isolation of tagged, newly synthesized proteins and their subsequent identification by MS. Recent advances in these techniques have furthered the investigation of translation at shorter timescales and at a greater depth of quantitative resolution, spurring a frenzy of research. Here, we review current methods to investigate nascent protein synthesis (Figure 1) and discuss their applications in the context of the central nervous system and in neurodegenerative diseases so far.

## 2. Metabolic Labeling of Nascent Peptides

Original methods to study translation began with metabolic labeling of polypeptide chains with radiolabeled methionine in 1953, a practice still used to validate changes in the overall levels of protein synthesis [21]. Currently, a more diverse toolkit enables researchers to choose from a variety of tags for incorporation. These techniques replace an endogenous molecule, such as methionine, with an isotopically or chemically altered version of the amino acid. The incorporation of these exogenous, tagged molecules into newly synthesized peptides allows for the affinity purification or alternative means of isolation of the peptides during MS analysis. By incorporating more than one tag at a time, researchers can also achieve multiplexed analyses across several experimental conditions, as discussed below.

### 2.1. Pulsed Stable Isotope Labeling with Amino Acids

Stable isotope labeling with amino acids in cell culture (SILAC), which was developed nearly two decades ago, provides a relatively simple technique to quantitatively measure proteins via MS [22]. This method involves the incorporation of isotopically labeled molecules, such as arginine or lysine, into newly synthesized proteins by replacing the endogenous supply of the molecule (Figure 1A). These new peptides can be differentiated by MS from pre-existing, unlabeled peptides due to the consistently altered mass of the tag. This technique was initially employed in cell cultures using timed pulses of isotopically labeled amino acids (pSILAC) [23,24]. By selectively ‘pulsing’ multiple isotopically labeled tags in the same sample, researchers can resolve *de novo* protein synthesis across conditions by tracking the incorporation of the different tags over time. For example, one can stably culture primary neurons under media containing a light isotope of arginine, induce membrane depolarization with a potassium chloride treatment, and in parallel supplement with heavy arginine-containing media for an hour. MS analysis of these samples with proper controls would exhibit the background levels of light arginine-incorporated proteins compared to newly translated, heavy arginine-incorporated proteins. Multiplexing with multiple amino acid isotopes is also possible, enabling the MS detection of nascent peptides across multiple timepoints or experimental conditions within the same sample.

Stable isotope incorporation is one of the most accurate techniques that assess protein synthesis in the living human brain, and it has recently been used to directly study translation in excised human cortical and hippocampal tissue [25]. However, to our knowledge, no study has coupled this technique with proteomics to detect newly synthesized proteins from the human brain. However, quickly after its initial development, SILAC was adapted for use in vivo to assess protein turnover and lifetime in the mouse brain [26]. This study identified striking differences in the regulation of protein turnover at the tissue, cellular, and protein complex level, highlighting important methodological considerations. For example, as the timescale of incorporation increases, proteins have an increased likelihood of being degraded. This results in a false decrease in signal even if the protein is effectively translated. This remains a potential confounder of analyses conducted at short timescales [27]. Since it was first described, the in vivo use of SILAC has mainly been limited to non-pulsed studies that have extensively mapped the dynamics of protein turnover in the brain in multiple species and in neurodegenerative models [28,29]. However, in vitro applications of pSILAC have flourished, expanding our understanding of protein translation and turnover rates across neuronal environments [30].

The Holt group recently published two articles utilizing pSILAC to assess changes in the axonal nascent proteome in Xenopus retinal ganglion cells [31,32]. In the first article, Cagnetta et al. identified over 350 proteins basally translated in retinal ganglion cell (RGC) axons and over 100 proteins differentially translated within just five minutes following the addition of axonal remodeling cues such as Sema3A [31]. This surprisingly short timescale was accomplished by pairing pSILAC with an ultrasensitive method of preparing samples for proteomics termed single pot solid-phase-enhanced sample preparation, or SP3, which considerably enhances the detection of tag-incorporated proteins [33]. Their more recent second study utilized 15 min of tag incubation with pSILAC-SP3 in RGC axons and demonstrated that Sema3A-dependent local protein synthesis is regulated by the protein kinase R-like endoplasmic reticulum kinase (PERK) phosphorylation of eukaryotic initiation factor 2 alpha (eIF2α), a subunit of the ternary translation complex which regulates translation initiation [32]. Since PERK is implicated in the pathology of multiple neurodegenerative diseases, this finding raises interesting connections between PERK, axonal translation, neural rewiring, and disease [34].

### 2.2. Non-Canonical Amino Acid Tagging (NCAT)

As an alternative to SILAC-based nascent proteomic experiments, the Schuman group developed a method in which synthetic, bio-orthogonal amino acids are incorporated into the proteome (Figure 1B) [35]. These methods use methionine analogs that are chemically and structurally altered but are still sterically similar enough to participate in normal translation [36]. These modifications enable downstream enrichment using click chemistry or affinity purification, a direct improvement over SILAC methods that increase MS detection [36]. The first use of bio-orthogonal noncanonical amino acid tagging (BONCAT) employed azidohomoalanine (AHA), an azide-bearing methionine analog, as a substitute for methionine in mammalian cell culture models [35]. With the added azide group, click chemistry allows for selective reactivity with alkyne-reacting agents, such as biotin species, which link the AHA-peptide with biotin to undergo affinity purification and MS ionization.

In the seminal study of BONCAT by Dieterich et al., human embryonic kidney (HEK) cells treated with AHA for two hours showed stable and reproducible incorporation into over 100 proteins which were confirmed as nascent peptides using pSILAC [36]. Since then, BONCAT has been developed into fluorescent non-canonical amino acids tagging (FUNCAT) for the visual imaging of protein synthesis using click chemistry techniques coupled with multicolor fluorescent alkyne-reactive molecules. FUNCAT has since been applied in a variety of neuronal contexts in vitro [37,38] and in vivo [39,40]). Importantly, FUNCAT can be used in parallel experiments with BONCAT to visualize the landscape of translation being purified and detected with BONCAT [41].

As MS and affinity purification techniques have progressed, BONCAT has grown to a remarkable depth of peptide capture across a variety of input samples. By combining BONCAT with CA1 neuropil micro-dissection, Hodas et al. identified the local dynamics of the neuropil nascent proteome following a dopamine receptor agonist treatment [42]. Bioinformatic analyses also revealed a candidate list of over 300 proteins involved in dopamine-mediated synaptic plasticity after just two and a half hours, which provides some of the first proteomic evidence of vast local protein synthesis in dendrites [42]. In 2016, Schanzenbächer et al. coupled BONCAT with synaptic up- or down-scaling agents in cultured hippocampal neurons to investigate the nascent proteins involved in the homeostatic remodeling of synapses [43]. Remarkably, nearly 80% (~6000 out of ~8000 proteins) of the hippocampal neuron proteome was synthesized within 24 h, and approximately 300 proteins were differentially translated depending on the type of synaptic scaling [43]. In 2018, Schanzenbächer and others expanded their previous work by assessing the changes in newly synthesized proteins at two hours in addition to 24 h of homeostatic scaling to discover rapid remodeling cues [44]. Compared to 24 h, only 168 proteins (nearly 35-fold less or ~2% of the total neuronal proteome) were differentially translated within two hours following synaptic remodeling cues. Remarkably, the functional categorization by gene ontology remained similar between timepoints [44]. To date, these reports are perhaps the deepest characterizations of the nascent synaptic proteome. However, the extent to which these differ from in vivo models of synaptic plasticity remains unknown.

As with pSILAC, shorter timescale proteomic analyses struggle to enrich sufficient labeled material for broad coverage in MS for in vivo use. In 2014, the Cline group developed a method to enrich biotin-tagged proteins from BONCAT-click chemistry experiments in vivo [45]. This method utilized the direct detection of biotin-containing tags (DiDBiT) which enabled Schiapparelli et al. to identify 20-fold more proteins compared to standard enrichment techniques, as nascently translated after just three hours of treatment with AHA [45]. The Cline group also published another in vivo application of BONCAT where they investigated the alterations in behavioral plasticity regulated by protein synthesis. Remarkably, their BONCAT experiments identified a single protein, cytoplasmic polyadenylation element binding protein (CPEB), whose acute synthesis following visual-conditioning regulated the link between translation and behavioral plasticity [46]. The Cline group went on to further utilize BONCAT to assess the nascent proteome following visual cues in Xenopus tadpoles, employing a shorter, five-hour incubation with AHA [47]. Here, Liu et al. identified over 5000 newly synthesized proteins overall and 80 candidate plasticity proteins that were differentially translated following visual cues [47]. Many of these novel candidate proteins have mammalian homologs and are reportedly implicated in neurodegenerative diseases [47]. By extensively characterizing and validating these results, these reports confirm the integral role of protein synthesis in experience-dependent plasticity in Xenopus and suggest similar mechanisms in mammals.

In mammalian systems, in vivo NCAT has primarily used methionine depletion followed by AHA or similar chemically-capturable methionine-analog pulsing to achieve the metabolic labeling of proteins. These techniques yield robust incorporation [48] without overtly disrupting murine development [49]. The first reported usage of in vivo AHA, termed pulsed azidohomoalanine labeling in mammals (PALM), utilized DiDBiT and reported the nascent translation of over 2800 proteins in the brain over several days of feeding [48]. While the total incorporation of AHA into nascent peptides was rather limited compared to intra-ocular injection or in vitro models, this study illustrated the success of non-invasive supplement with AHA via feeding. McClatchy and others in 2015 further developed this technique and showed—via subcellular fractionation into nuclear, mitochondrial, and synaptic fractions—that AHA-labeled proteins were retained. These data suggest that this method can also be used to identify compartment-specific protein synthesis alterations [48]. Recently, the Götz group utilized intraperitoneal injections of AHA to label the *de novo* proteome in multiple mouse models of tauopathy within four hours of treatment [50], recapitulating our earlier results that mutant tau selectively alters the synthesis of ribosomal proteins in vivo [51]. Together, these results demonstrate the ability for NCAT to identify alterations in protein synthesis in vivo across a multitude of disease models.

### 2.3. Combined BONCAT and SILAC (BONLAC)

To better assess nascent protein translation compared to steady-state protein levels, researchers combined the BONCAT and SILAC techniques together to form BONLAC (Figure 1C) [52]. This method, originally tested using HEK293 cells, was adapted for use in acute hippocampal slices in 2016 by Klann and others [41]. Here, Bowling et al. first optimized the technique for AHA concentration and duration of treatment and confirmed the presence of a minimal nonspecific signal which was not dependent on active protein synthesis. They found that a four-hour treatment with AHA and stable arginine and lysine isotopes labeled over 2000 proteins that were detected reproducibly by MS. Then, they assessed nascent translation differences after treatment with brain-derived neurotrophic factor (BDNF) and validated several novel candidate proteins that were differentially translated [41]. Further comparisons identified that the effect of BDNF varies significantly between the nascent proteome in ex vivo slices and hippocampal neuronal cultures, further emphasizing the need for nascent proteomic methods in vivo [41].

The Klann group recently followed up on their study by using BONLAC to assess the nascent proteome differences in acute slices prepared from fragile X syndrome (FXS) model mice, which do not express Fmr1, or fragile X mental retardation 1 [53]. Here, Bowling et al. identified over 300 consistently altered proteins in FXS hippocampal slices and validated the technique against three potential candidate proteins involved in synaptic signaling. Surprisingly, many of the differentially translated proteins were involved in metabolism-specific responses, providing strong evidence for metabolism-specific alterations in FXS [53]. They further probed for alterations in glutamate receptor activation-induced protein synthesis in FXS by combining BONLAC with a glutamate receptor mGluR1/5 agonist DHPG treatment. They identified consistently altered proteins across these treatments and subsequently validated two of them as potential blood-based biomarkers for FXS in humans [53]. While BONLAC seems to be limited to ex vivo uses so far, behavioral tasks and other analyses of complex brain function may be coupled with downstream slice preparation. Regardless, BONLAC provides a greater detection of newly synthesized proteins within a four-hour window in disease models, even though the heightened sensitivity comes with an increased cost and difficulty of analysis when compared to BONCAT-based methods. Importantly, other combination methods have also been reported, such as utilizing different proteomic sample preparations or loading (QuanCAT [54]) and heavy isotope AHA (HILAQ [55,56]), though these are limited to in vitro culture.

### 2.4. Mutated MetRS-BONCAT

Though NCATs enable the greater interrogation of translation in neurological contexts, a limiting factor is the poor sensitivity inherent in using non-canonical forms of methionine results in nearly 500 times lower incorporation compared to regular methionine [57]. Reports quickly emerged where the genetic code was expanded to include tRNAs capable of incorporating non-canonical amino acids to assess nascent translation in vitro [58]. In 2015, Erdmann et al. further adapted this technique in vivo in Drosophila by expressing a mutated methionyl-tRNA synthetase (mMetRS) in place of the endogenous enzyme (Figure 1D). This enabled the cell-specific labelling of nascent proteins [59]. The leucine to glycine mutation in the methionyl-tRNA binding pocket facilitated the non-canonical amino acid incorporation of azidonorleucine (ANL), increasing the rate of incorporation and subsequent detection by MS [59]. In 2017, the Schuman group expanded this cell-type specific nascent proteomics method for use in mice for the first time [60,61]. Alvarez-Castelao and others identified over 2500 newly synthesized proteins tagged with ANL following 21 days of treatment in excitatory hippocampal neurons, including many disease-associated proteins [60]. They continued to identify neuronal type-specific nascent proteomes and over 200 novel proteins which are differentially translated in hippocampal excitatory neurons shortly following exposure to a novel environment [60]. The Chin group later reported the usage of viral-mediated expression of orthogonal tRNA synthetases to couple cell-type specific and region-specific nascent proteomics in mouse brain [62]. To date, no report has systematically compared these methods within the same model or cell type. However, overall, cell-specific nascent proteomics provides immense potential to unravel the dynamics of translation and protein turnover in response to cellular stress or disease. For example, recent reports have identified a specific vulnerability of cortical excitatory neurons in Alzheimer’s disease [63]. By using mMetRS expressed in these cortical excitatory neurons, researchers may investigate whether altered translational dynamics are involved in this vulnerability.

## 3. Puromycin Incorporation into Polypeptides

Puromycin is an aminonucleoside antibiotic analog of tyrosine-tRNA that irreversibly binds to the C-terminus of a polypeptide, terminating synthesis for that protein [64]. Initial studies investigating protein translation and memory used puromycin at high concentrations (450 mg/kg) for two hours to inhibit translation [1]. As puromycin readily penetrates not only the blood-brain barrier but also cell membranes, methods using puromycin to tag actively elongating proteins emerged in 2005 in a technique termed SUnSET, or surface sensing of translation (Figure 1E) [65]. Here, Schmidt and others reported the first use of anti-puromycin antibodies to selectively detect changes in puromycinylated protein levels, an analog for the amount of proteins translated during puromycin treatment [65]. While this method enables the timely and less expensive detection of translation as compared to genetic manipulation or costly non-canonical amino acid treatments, it has several drawbacks that limit its usage. Importantly, puromycinylated proteins are rapidly degraded [66], and, at high concentrations, puromycin inhibits eukaryotic protein synthesis, greatly limiting the timescale potential for analysis [1]. However, empirically optimizing conditions can limit these effects, and puromycin has now been extensively used as a nascent protein tag in primary neuronal culture [67], in ex vivo hippocampal slice culture [68], and even in vivo [51]. In our recent study, we utilized a 30-min intraperitoneal injection of puromycin (225 mg/kg) followed by antibody affinity capture coupled with MS in the first usage of in vivo nascent proteomics in a mouse model of neurodegeneration [51]. We had previously attempted intracranial injections, which resulted in inconsistent incorporation. However, intraperitoneal injections offered the stable puromycinylation of nascent proteins. Our experiments identified a putative mechanism behind the decreased synthesis of ribosomal proteins in tauopathy, which has since been supported by another study from the Götz lab utilizing BONCAT, thereby validating in vivo puromycin incorporation as a means to assess nascent translation [50]. An advantage of puromycin is that it incorporates into every cell indiscriminately, thus offering whole brain analysis; however, it can be coupled with bio-informatic analyses to identify proteins expressed in unique cell types.

Other methods utilizing puromycin-based nascent protein capture include treatment with puromycin analogs, which are amenable to click chemistry or fluorescent-based approaches [69,70]. However, these altered puromycin analogs exhibit reduced transport into cells, do not readily pass the blood brain barrier, and have only been established to effectively tag nascent proteins for MS capture in vitro or in organoid systems [71]. Lastly, puromycin-based nascent protein tagging has been accomplished in cellular and tissue lysate by first isolating translating polysomes and then treating with puromycin conjugated with biotin, allowing subsequent capture and analysis via MS in a technique termed puromycin-associated nascent chain proteomics (PUNCH-P) (Figure 1F) [72]. Though this technique allows for a greater temporal resolution than typical pSILAC or NCAT methods, it has the limitation of requiring polysomal isolation, which may introduce a bias of the detected protein pool. Indeed, PUNCH-P has reported a small bias toward lower molecular weight proteins, though this limitation may be minimized considering the nearly 10-fold higher amounts of nascent proteins detected using this technique at a considerably reduced cost [70]. Though initially utilized in 2013 by the Elroy-Stein group to characterize the first translational profile of the developing mouse brain, PUNCH-P has yet to be used to analyze translation in the diseased brain [72].

## 4. Technical Considerations

While the duration of tag incubation can be tailored to investigate translational dynamics across a variety of temporal ranges from several minutes to several hours, it is important to also consider the methodological limitations for the nascent proteomic techniques described here. As further development enhances the resolution of nascent proteomics to shorter timescales, reproducibility becomes a greater concern, both due to limitations in the technique and in the possible inherent biological variance. Experimentally determined Pearson’s correlation coefficients (PCC) between replicates are very high (>0.95) in medium (one to six hours) and long (over six hours) incubation times. However, short durations (under one hour) of tag incubation potentially suffer from technical variability stemming from differences in tag availability across cell compartments as well as fundamental kinetic differences between the exogenous, tagged amino acid and the endogenous version. As these sources of variation cause differences in tag incorporation, these would introduce experimental bias detected by downstream MS. For example, the shortest tag incubation time described in this review used a five-minute pSILAC incubation window coupled with SP3 [31]. The experimental PCC was 0.60 with three replicates, which the authors noted was higher than a previously reported BONLAC experiment [31]. This limited consistency may provide type I (false positive) or type II (false negative) errors. As the kinetic properties of these tags have not been extensively detailed in complex samples such as brain tissue, experiments assessing rapid translational dynamics using these techniques should attempt to minimize variability where possible, increase the number of replicates used, and validate any identified targets.

## 5. Conclusions

Translation is a crucial component of neuronal health as well as memory and cognition, and it has increasingly been implicated in neurodegenerative diseases. However, little is known about how translational dynamics contribute to neuronal function. We briefly discussed a variety of methods (Table 1) developed over the last decade that prime the field to detect quantitative changes in the nascent proteome, both in vitro and in vivo. Thus far, only a few studies have utilized these methods to discern alterations in protein synthesis in the diseased mammalian brain. While promising, there are many unexplored avenues using nascent proteomics to assess altered translation across developmental stages, environmental cues, and diseases. With more rapid timescale detection, single cell-type specificity, and broad proteomic coverage, these methods offer a deeper understanding of neurobiological mechanisms governed by translation and the development of novel therapeutic targets.

## Figures and Tables

**Table 1:** Comparison of proteomic techniques that focus on the nascent proteome and experimental considerations.

	Methodology	Incubation Time	Considerations	Current Model Utility	Refs.
pSILAC: Pulsed Stable Isotopic Labeling of Amino acids	Pulsed isotopic labeling of amino acids	Short to Long	Robust incorporation but generally requires long incubation times. May introduce a bias of tag incorporation.	In vitro	[31,32]
BONCAT: Bio-orthogonal Non-Canonical Amino acid Tagging	Non-canonical amino acid incorporation and chemical capture	Short to Medium	Weak incorporation at shorter incubation timescales. Can be adapted for fluorescent detection. Strong MS detection after purification.	In vitro Ex vivo In vivo	[35,36,37,38,39,40,41,42,43,44,45,46,47,49,50]
PALM: Pulsed Azidohomoalanine Labeling in Mammals	In vivo BONCAT using AHA-enriched feed	Long	Weak incorporation and requires multi-day diet on enriched feed. Nascent translation can be detected in sub-cellular fractions.	In vivo	[48]
BONLAC: Combinatorial BONCAT and pSILAC	Combined pSILAC with BONCAT enrichment	Medium	Enables the robust detection of nascent peptides but with a greater experimental complexity.	In vitro Ex vivo	[41,52,53]
mMetRS BONCAT: Mutated Methionyl-tRNA synthetase coupled with BONCAT	BONCAT but with cell-specific expression of expanded tRNAs	Medium	Requires genetic manipulation or viral-mediated genetic transfer but can be adapted for cell-specific investigations of nascent translation.	In vitro In vivo	[59,60,61,62]
Puromycin	Puromycin labeling and affinity capture	Short	Requires simple injection followed by affinity capture. Can inhibit translation at high concentrations.	In vitro Ex vivo In vivo	[51,65,67,68]
PUNCH-P: Puromycin associated Nascent Chain Proteomics	Puromycin-biotin labeling and chemical capture	Short	Requires tissue homogenization prior to incubation but with strong incorporation.	In vitro In vivo^&^	[70,72]

Experimental incubation times range can be short (five minutes to one hour), medium (over one hour to six hours), or long (over six hours). The current model utility describes the sample conditions used in nascent proteomic techniques discussed in this review. Techniques with in vivo utility denote previously published studies where the chosen tag was incorporated into nascent proteins in live animals for downstream analysis. ^&^ PUNCH-P requires tissue homogenization prior to puromycin-biotin incorporation for in vivo use.

## References

[1] Flexner J.B., Flexner L.B., Stellar E. (1963). Memory in mice as affected by intracerebral puromycin. Science.

[2] Hernandez P.J., Abel T. (2008). The role of protein synthesis in memory consolidation: Progress amid decades of debate. Neurobiol. Learn. Mem..

[3] Jarome T.J., Helmstetter F.J. (2014). Protein degradation and protein synthesis in long-term memory formation. Front. Mol. Neurosci..

[4] Abraham W.C., Williams J.M. (2008). LTP maintenance and its protein synthesis-dependence. Neurobiol. Learn. Mem..

[5] Buffington S.A., Huang W., Costa-Mattioli M. (2014). Translational control in synaptic plasticity and cognitive dysfunction. Annu. Rev. Neurosci..

[6] Kapur M., Monaghan C.E., Ackerman S.L. (2017). Regulation of mRNA Translation in Neurons-A Matter of Life and Death. Neuron.

[7] Hafner A.S., Donlin-Asp P.G., Leitch B., Herzog E., Schuman E.M. (2019). Local protein synthesis is a ubiquitous feature of neuronal pre- and postsynaptic compartments. Science.

[8] Khalil B., Morderer D., Price P.L., Liu F., Rossoll W. (2018). mRNP assembly, axonal transport, and local translation in neurodegenerative diseases. Brain Res..

[9] Kim E., Jung H. (2015). Local protein synthesis in neuronal axons: Why and how we study. BMB Rep..

[10] Taylor A.M., Wu J., Tai H.C., Schuman E.M. (2013). Axonal translation of beta-catenin regulates synaptic vesicle dynamics. J. Neurosci..

[11] Costa-Mattioli M., Sossin W.S., Klann E., Sonenberg N. (2009). Translational control of long-lasting synaptic plasticity and memory. Neuron.

[12] Cracco J.B., Serrano P., Moskowitz S.I., Bergold P.J., Sacktor T.C. (2005). Protein synthesis-dependent LTP in isolated dendrites of CA1 pyramidal cells. Hippocampus.

[13] Huber K.M., Kayser M.S., Bear M.F. (2000). Role for rapid dendritic protein synthesis in hippocampal mGluR-dependent long-term depression. Science.

[14] Saxena S., Caroni P. (2011). Selective neuronal vulnerability in neurodegenerative diseases: From stressor thresholds to degeneration. Neuron.

[15] Scarnati M.S., Kataria R., Biswas M., Paradiso K.G. (2018). Active presynaptic ribosomes in the mammalian brain, and altered transmitter release after protein synthesis inhibition. Elife.

[16] Ping L., Duong D.M., Yin L., Gearing M., Lah J.J., Levey A.I., Seyfried N.T. (2018). Global quantitative analysis of the human brain proteome in Alzheimer’s and Parkinson’s Disease. Sci. Data.

[17] Iwasaki S., Ingolia N.T. (2017). The Growing Toolbox for Protein Synthesis Studies. Trends Biochem. Sci..

[18] Ingolia N.T., Lareau L.F., Weissman J.S. (2011). Ribosome profiling of mouse embryonic stem cells reveals the complexity and dynamics of mammalian proteomes. Cell.

[19] Pircher A., Gebetsberger J., Polacek N. (2014). Ribosome-associated ncRNAs: An emerging class of translation regulators. RNA Biol..

[20] Wilson R.S., Nairn A.C. (2018). Cell-Type-Specific Proteomics: A Neuroscience Perspective. Proteomes.

[21] Smellie R.M., Mc I.W., Davidson J.N. (1953). The incorporation of 15N, 35S and 14C into nucleic acids and proteins of rat liver. Biochim. Biophys. Acta.

[22] Ong S.E., Blagoev B., Kratchmarova I., Kristensen D.B., Steen H., Pandey A., Mann M. (2002). Stable isotope labeling by amino acids in cell culture, SILAC, as a simple and accurate approach to expression proteomics. Mol. Cell Proteom..

[23] Schwanhausser B., Gossen M., Dittmar G., Selbach M. (2009). Global analysis of cellular protein translation by pulsed SILAC. Proteomics.

[24] Selbach M., Schwanhausser B., Thierfelder N., Fang Z., Khanin R., Rajewsky N. (2008). Widespread changes in protein synthesis induced by microRNAs. Nature.

[25] Smeets J.S.J., Horstman A.M.H., Schijns O., Dings J.T.A., Hoogland G., Gijsen A.P., Goessens J.P.B., Bouwman F.G., Wodzig W., Mariman E.C. (2018). Brain tissue plasticity: Protein synthesis rates of the human brain. Brain.

[26] Price J.C., Guan S., Burlingame A., Prusiner S.B., Ghaemmaghami S. (2010). Analysis of proteome dynamics in the mouse brain. Proc. Natl. Acad. Sci. USA.

[27] McShane E., Sin C., Zauber H., Wells J.N., Donnelly N., Wang X., Hou J., Chen W., Storchova Z., Marsh J.A. (2016). Kinetic Analysis of Protein Stability Reveals Age-Dependent Degradation. Cell.

[28] Fornasiero E.F., Mandad S., Wildhagen H., Alevra M., Rammner B., Keihani S., Opazo F., Urban I., Ischebeck T., Sakib M.S. (2018). Precisely measured protein lifetimes in the mouse brain reveal differences across tissues and subcellular fractions. Nat. Commun..

[29] Xu P., Tan H., Duong D.M., Yang Y., Kupsco J., Moberg K.H., Li H., Jin P., Peng J. (2012). Stable isotope labeling with amino acids in Drosophila for quantifying proteins and modifications. J. Proteome Res..

[30] Dorrbaum A.R., Kochen L., Langer J.D., Schuman E.M. (2018). Local and global influences on protein turnover in neurons and glia. Elife.

[31] Cagnetta R., Frese C.K., Shigeoka T., Krijgsveld J., Holt C.E. (2018). Rapid Cue-Specific Remodeling of the Nascent Axonal Proteome. Neuron.

[32] Cagnetta R., Wong H.H., Frese C.K., Mallucci G.R., Krijgsveld J., Holt C.E. (2019). Noncanonical Modulation of the eIF2 Pathway Controls an Increase in Local Translation during Neural Wiring. Mol. Cell.

[33] Hughes C.S., Foehr S., Garfield D.A., Furlong E.E., Steinmetz L.M., Krijgsveld J. (2014). Ultrasensitive proteome analysis using paramagnetic bead technology. Mol. Syst. Biol..

[34] Bell M.C., Meier S.E., Ingram A.L., Abisambra J.F. (2016). PERK-opathies: An Endoplasmic Reticulum Stress Mechanism Underlying Neurodegeneration. Curr. Alzheimer Res..

[35] Dieterich D.C., Lee J.J., Link A.J., Graumann J., Tirrell D.A., Schuman E.M. (2007). Labeling, detection and identification of newly synthesized proteomes with bioorthogonal non-canonical amino-acid tagging. Nat. Protoc..

[36] Dieterich D.C., Link A.J., Graumann J., Tirrell D.A., Schuman E.M. (2006). Selective identification of newly synthesized proteins in mammalian cells using bioorthogonal noncanonical amino acid tagging (BONCAT). Proc. Natl. Acad. Sci. USA.

[37] Dieterich D.C., Hodas J.J., Gouzer G., Shadrin I.Y., Ngo J.T., Triller A., Tirrell D.A., Schuman E.M. (2010). In situ visualization and dynamics of newly synthesized proteins in rat hippocampal neurons. Nat. Neurosci..

[38] Tcherkezian J., Brittis P.A., Thomas F., Roux P.P., Flanagan J.G. (2010). Transmembrane receptor DCC associates with protein synthesis machinery and regulates translation. Cell.

[39] Hinz F.I., Dieterich D.C., Schuman E.M. (2013). Teaching old NCATs new tricks: Using non-canonical amino acid tagging to study neuronal plasticity. Curr. Opin. Chem. Boil..

[40] Liu H.H., Cline H.T. (2016). Fragile X Mental Retardation Protein Is Required to Maintain Visual Conditioning-Induced Behavioral Plasticity by Limiting Local Protein Synthesis. J. Neurosci..

[41] Bowling H., Bhattacharya A., Zhang G., Lebowitz J.Z., Alam D., Smith P.T., Kirshenbaum K., Neubert T.A., Vogel C., Chao M.V. (2016). BONLAC: A combinatorial proteomic technique to measure stimulus-induced translational profiles in brain slices. Neuropharmacology.

[42] Hodas J.J., Nehring A., Hoche N., Sweredoski M.J., Pielot R., Hess S., Tirrell D.A., Dieterich D.C., Schuman E.M. (2012). Dopaminergic modulation of the hippocampal neuropil proteome identified by bioorthogonal noncanonical amino acid tagging (BONCAT). Proteomics.

[43] Schanzenbacher C.T., Sambandan S., Langer J.D., Schuman E.M. (2016). Nascent Proteome Remodeling following Homeostatic Scaling at Hippocampal Synapses. Neuron.

[44] Schanzenbacher C.T., Langer J.D., Schuman E.M. (2018). Time- and polarity-dependent proteomic changes associated with homeostatic scaling at central synapses. Elife.

[45] Schiapparelli L.M., McClatchy D.B., Liu H.H., Sharma P., Yates J.R., Cline H.T. (2014). Direct detection of biotinylated proteins by mass spectrometry. J. Proteome Res..

[46] Shen W., Liu H.H., Schiapparelli L., McClatchy D., He H.Y., Yates J.R., Cline H.T. (2014). Acute synthesis of CPEB is required for plasticity of visual avoidance behavior in Xenopus. Cell Rep..

[47] Liu H.H., McClatchy D.B., Schiapparelli L., Shen W., Yates J.R., Cline H.T. (2018). Role of the visual experience-dependent nascent proteome in neuronal plasticity. Elife.

[48] McClatchy D.B., Ma Y., Liu C., Stein B.D., Martinez-Bartolome S., Vasquez D., Hellberg K., Shaw R.J., Yates J.R. (2015). Pulsed Azidohomoalanine Labeling in Mammals (PALM) Detects Changes in Liver-Specific LKB1 Knockout Mice. J. Proteome Res..

[49] Calve S., Witten A.J., Ocken A.R., Kinzer-Ursem T.L. (2016). Incorporation of non-canonical amino acids into the developing murine proteome. Sci. Rep..

[50] Evans H.T., Benetatos J., Van Roijen M., Bodea L.G., Gotz J. (2019). Decreased synthesis of ribosomal proteins in tauopathy revealed by non-canonical amino acid labelling. EMBO J..

[51] Koren S.A., Hamm M.J., Meier S.E., Weiss B.E., Nation G.K., Chishti E.A., Arango J.P., Chen J., Zhu H., Blalock E.M. (2019). Tau drives translational selectivity by interacting with ribosomal proteins. Acta Neuropathol..

[52] Zhang G., Bowling H., Hom N., Kirshenbaum K., Klann E., Chao M.V., Neubert T.A. (2014). In-depth quantitative proteomic analysis of de novo protein synthesis induced by brain-derived neurotrophic factor. J. Proteome Res..

[53] Bowling H., Bhattacharya A., Zhang G., Alam D., Lebowitz J.Z., Bohm-Levine N., Lin D., Singha P., Mamcarz M., Puckett R. (2019). Altered steady state and activity-dependent de novo protein expression in fragile X syndrome. Nat. Commun..

[54] Howden A.J., Geoghegan V., Katsch K., Efstathiou G., Bhushan B., Boutureira O., Thomas B., Trudgian D.C., Kessler B.M., Dieterich D.C. (2013). QuaNCAT: Quantitating proteome dynamics in primary cells. Nat. Methods.

[55] Ma Y., McClatchy D.B., Barkallah S., Wood W.W., Yates J.R. (2018). Quantitative analysis of newly synthesized proteins. Nat. Protoc.

[56] Ma Y., McClatchy D.B., Barkallah S., Wood W.W., Yates J.R. (2017). HILAQ: A Novel Strategy for Newly Synthesized Protein Quantification. J. Proteome Res..

[57] Beatty K.E., Liu J.C., Xie F., Dieterich D.C., Schuman E.M., Wang Q., Tirrell D.A. (2006). Fluorescence visualization of newly synthesized proteins in mammalian cells. Angew. Chem. Int. Ed..

[58] Ngo J.T., Schuman E.M., Tirrell D.A. (2013). Mutant methionyl-tRNA synthetase from bacteria enables site-selective N-terminal labeling of proteins expressed in mammalian cells. Proc. Natl. Acad. Sci. USA.

[59] Erdmann I., Marter K., Kobler O., Niehues S., Abele J., Muller A., Bussmann J., Storkebaum E., Ziv T., Thomas U. (2015). Cell-selective labelling of proteomes in Drosophila melanogaster. Nat. Commun..

[60] Alvarez-Castelao B., Schanzenbacher C.T., Hanus C., Glock C., Tom Dieck S., Dorrbaum A.R., Bartnik I., Nassim-Assir B., Ciirdaeva E., Mueller A. (2017). Cell-type-specific metabolic labeling of nascent proteomes in vivo. Nat. Biotechnol..

[61] Alvarez-Castelao B., Schanzenbacher C.T., Langer J.D., Schuman E.M. (2019). Cell-type-specific metabolic labeling, detection and identification of nascent proteomes in vivo. Nat. Protoc..

[62] Krogager T.P., Ernst R.J., Elliott T.S., Calo L., Beranek V., Ciabatti E., Spillantini M.G., Tripodi M., Hastings M.H., Chin J.W. (2018). Labeling and identifying cell-specific proteomes in the mouse brain. Nat. Biotechnol..

[63] Fu H., Possenti A., Freer R., Nakano Y., Villegas N.C.H., Tang M., Cauhy P.V.M., Lassus B.A., Chen S., Fowler S.L. (2019). A tau homeostasis signature is linked with the cellular and regional vulnerability of excitatory neurons to tau pathology. Nat. Neurosci..

[64] Nathans D. (1964). Puromycin Inhibition of Protein Synthesis: Incorporation of Puromycin into Peptide Chains. Proc. Natl. Acad. Sci. USA.

[65] Schmidt E.K., Clavarino G., Ceppi M., Pierre P. (2009). SUnSET, a nonradioactive method to monitor protein synthesis. Nat. Methods.

[66] Prouty W.F., Goldberg A.L. (1972). Fate of abnormal proteins in E. coli accumulation in intracellular granules before catabolism. Nat. New Biol..

[67] Meier S., Bell M., Lyons D.N., Rodriguez-Rivera J., Ingram A., Fontaine S.N., Mechas E., Chen J., Wolozin B., LeVine H. (2016). Pathological Tau Promotes Neuronal Damage by Impairing Ribosomal Function and Decreasing Protein Synthesis. J. Neurosci..

[68] Hoeffer C.A., Santini E., Ma T., Arnold E.C., Whelan A.M., Wong H., Pierre P., Pelletier J., Klann E. (2013). Multiple components of eIF4F are required for protein synthesis-dependent hippocampal long-term potentiation. J. Neurophysiol..

[69] Starck S.R., Green H.M., Alberola-Ila J., Roberts R.W. (2004). A general approach to detect protein expression in vivo using fluorescent puromycin conjugates. Chem. Biol..

[70] Aviner R., Geiger T., Elroy-Stein O. (2014). Genome-wide identification and quantification of protein synthesis in cultured cells and whole tissues by puromycin-associated nascent chain proteomics (PUNCH-P). Nat. Protoc..

[71] Jose L.H.S., Signer R.A.J. (2019). Cell-type-specific quantification of protein synthesis in vivo. Nat. Protoc..

[72] Aviner R., Geiger T., Elroy-Stein O. (2013). Novel proteomic approach (PUNCH-P) reveals cell cycle-specific fluctuations in mRNA translation. Genes Dev..

